# ARAF regulates malignant progression of bladder cancer through the p38MAPK pathway

**DOI:** 10.1515/med-2026-1422

**Published:** 2026-05-26

**Authors:** Xian Zhao, Xiaojing Xu

**Affiliations:** Department of Urology, Taikang Tongji (Wuhan) Hospital, Wuhan, China; Health Care Office, Wuhan No. 1 Hospital (Wuhan Hospital of Traditional Chinese and Western Medicine), Wuhan, China

**Keywords:** bladder cancer, ARAF, apoptosis, p38MAPK pathway

## Abstract

**Objectives:**

Bioinformatics analysis suggests that the A-Raf proto-oncogene serine/threonine kinase (ARAF) is closely associated with bladder cancer and the mitogen-activated protein kinase (MAPK) pathway. In this study, we aimed to investigate the role and molecular mechanisms of ARAF in bladder cancer.

**Methods:**

Genes associated with bladder cancer were analyzed using public databases, and KEGG analysis was performed. ARAF expression in bladder cancer cells was assessed using RT-qPCR and western blotting. Cell proliferation and apoptosis were measured using EdU fluorescence and flow cytometry, respectively. Cell migration and invasion were detected using Transwell assays. EMT-related and p38MAPK pathway proteins were analyzed through western blotting. Finally, the p38 MAPK pathway activator, anisomycin, was used to explore the mechanism of ARAF in cancer cells.

**Results:**

Intersecting genes related to bladder cancer were identified from the GeneCards and CTD databases, and ARAF was enriched in the p38MAPK pathway. ARAF expression was elevated in cancer cells, and its inhibition significantly reduced cell proliferation and metastasis, promoted cell apoptosis, and suppressed p38MAPK pathway activation. Anisomycin reversed the inhibitory effects of si-ARAF on T24 cells.

**Conclusions:**

Inhibition of ARAF suppresses malignant proliferation and metastasis of bladder cancer cells by repressing the p38MAPK pathway.

## Introduction

Bladder cancer is a malignant tumor originating from the mucosal epithelium of the bladder and is one of the most common malignancies of the urinary system [1]. Its harm lies not only in the impairment of urinary function but also in its potential to threaten life through metastasis [2]. Bladder cancer has a high global incidence, with approximately 82,000 new cases and 16,000 deaths reported in the United States in 2023, making it the fourth most common cancer in men [3]. Data from China in 2022 data showed an incidence rate of 9.3 per 100,000 people and a mortality rate of 4.1 per 100,000 people, with the urban incidence rate approximately 2.5 times higher than that in rural areas [4]. The incidence of bladder cancer is significantly higher in men than in women, with a male-to-female ratio of approximately 3:1–4:1, which may be related to factors such as androgen-mediated promotion of oncogene expression, smoking, and occupational exposures [5]. In addition, the high recurrence rate and treatment complexity of bladder cancer impose a substantial public health burden [6]. Therefore, the development of novel diagnostic and therapeutic targets for bladder cancer is urgently needed.

A-Raf proto-oncogene serine/threonine kinase (ARAF), a member of the RAF kinase family, is located on chromosome 15q14 and regulates key biological processes such as cell proliferation, differentiation, survival, and apoptosis [7], [8], [9]. ARAF inhibits ERBB3 promoter activity and regulates ERBB3 expression through the transcription factor KLF5, thereby suppressing lung cancer metastasis [10]. ARAF expression was found to be upregulated in gallbladder cancer tissues, and silencing ARAF effectively inhibited gallbladder cancer cell proliferation and xenograft tumor growth, suggesting that ARAF may serve as a potential therapeutic target as an oncogene in gallbladder cancer [11]. In addition, ARAF mutations were found in 11 % of 107 patients with intrahepatic cholangiocarcinoma (iCCA), suggesting that ARAF may be a therapeutic target for iCCA [12]. Notably, a recent case report showed aberrant ARAF expression in a patient with bladder cancer [13]. Although the role of ARAF has been documented in several tumor types, its expression pattern, clinical significance, and biological function in bladder cancer remain largely unclear. Notably, analysis of public genomic datasets, including the cancer genome atlas (TCGA) and gene expression omnibus (GEO) databases, has revealed aberrant expression of ARAF in bladder cancer tissues compared with normal bladder epithelium. Moreover, elevated ARAF expression has been associated with advanced tumor stage, high histological grade, and poor clinical outcomes in bladder cancer patients. These clinical and genomic observations strongly suggest that ARAF may serve as a functionally important regulator and a plausible therapeutic target in bladder cancer. However, the specific role and underlying mechanism of ARAF in bladder cancer progression have not been systematically investigated. However, the specific role and underlying mechanisms of ARAF in bladder cancer remain unclear.

The p38 mitogen-activated protein kinase (MAPK) pathway is a classical intracellular pathway that is widely involved in physiological and pathological processes, including cellular stress responses, inflammation, apoptosis, differentiation, and cell cycle regulation [14], [15], [16]. This pathway is usually activated by external stress stimuli and plays a key role in immune regulation and disease development [17]. A study showed that, by establishing a model to isolate breast cancer stem cells and performing transcriptome analysis, blockade of the p38 MAPK pathway effectively reduced the motility and metastatic capacity of breast cancer stem cells, suggesting that the p38 MAPK signaling pathway could be a potential therapeutic target for breast cancer [18]. In addition, a previous study demonstrated that tectoridin (TEC) inhibited colon cancer progression by downregulating the p38 MAPK pathway [19]. Although several studies have confirmed that the p38 MAPK pathway is involved in regulating the malignant progression of bladder cancer [20], [21], [22], whether ARAF regulates bladder cancer malignancy through the p38 MAPK pathway remains unclear.

In summary, we aimed to investigate the role of ARAF in bladder cancer cells and to determine whether it influences malignant proliferation and metastasis by regulating the p38 MAPK pathway.

## Materials and methods

### Cell culture and treatment

The human ureter epithelial immortalized cell line SV-HUC-1 (CL-0222) and human bladder cancer cell lines T24 (CL-0227) and J82 (CL-0125) were purchased from Wuhan Procell Biological Company. Cells were cultured in Dulbecco’s Modified Eagle Medium (DMEM, C2701, Beyotime) supplemented with 10 % fetal bovine serum (FBS; 164210, Procell) and 1 % penicillin-streptomycin (PB180120, Procell), and maintained in a humidified incubator at 37 °C with 5 % CO_2_.

To induce p38 activity, cells were treated with 5 μM anisomycin (HY-18982, MCE) for 24 h according to previous study [23].

### Public databases

The GeneCards (https://www.genecards.org/) and CTD databases (https://ctdbase.org/) were searched using the term “bladder cancer,” and genes associated with bladder cancer were retrieved. Intersecting genes were identified by constructing a Venn diagram using an online tool (https://bioinfogp.cnb.csic.es/tools/venny/). These genes were then imported into the KOBAS tool (http://bioinfo.org/kobas/genelist/) for KEGG pathway analysis.

### Cell transfection

To exogenously regulate ARAF expression in cancer cells, siRNA targeting for ARAF was synthesized. All siRNA sequences were synthesized by Anhui General Biologicals. The si-ARAF and si-NC sequences were as follows: si-ARAF: SS Sequence: 5′-GGA​AGA​CGC​GAC​AUG​UCA​ACA-3′, AS sequence: 5′-UUG​ACA​UGU​CGC​GUC​UUC​CUG-3′. si-NC: 5′-GTT​CTC​CGA​ACG​TGT​CAC​GT-3′. All sequences were transfected into cells using Lipo6000 transfection reagent (C0526, Beyotime), and subsequent experiments were performed 48 h after transfection.

### 5-Ethynyl-2′-deoxyuridine (EdU) assay

The EdU assay was performed according to the instructions of the EdU kit (G1601, Servicebio). Briefly, treated cells were washed two to three times with PBS, incubated with pre-configured EdU working solution at 37 °C for 2 h, and then fixed and permeabilized. The pre-configured EdU click reaction solution was then added and incubated for 30 min at room temperature in the dark. After washing with PBS two to three times, nuclei were stained with DAPI (C1006, Beyotime). Finally, cells were observed using a fluorescence microscope (Eclipse Ci-L, Nikon).

### Annexin V-FITC/PI double staining

Apoptosis was assessed according to the manufacturer’s instructions (556547, BD Biosciences). Briefly, treated cells from different groups were digested with EDTA-free trypsin. After washing with PBS, cells were incubated with 500 μL binding buffer containing 10 μL Annexin V-FITC for 30 min at room temperature in the dark. Subsequently, 5 μL PI solution was added, followed by incubation for 30 min at 37 °C in the dark. Finally, 500 μL PBS was added to resuspend the cells, and apoptosis was analyzed using flow cytometry.

### Transwell assay

Cell migration and invasion were assessed using Transwell chambers with an 8 μm pore size (3422, Corning). For migration assays, transfected cells were digested, resuspended in serum-free medium, and seeded into the upper chambers. Medium containing serum (800 μL) was added to the lower chambers, and the plates were incubated for 24 h. For invasion assays, the upper membranes were precoated with Matrigel (C0371, Beyotime). Transfected cells were then digested, resuspended, and seeded into the upper chambers, while 800 μL of serum-containing medium was added to the lower chambers. The chambers were incubated for 24 h. After incubation, cells on the membranes were fixed with 4 % paraformaldehyde for 30 min at room temperature and stained with crystal violet solution (C0121, Beyotime). Non-migrated or non-invaded cells on the upper surface of the membranes were carefully removed with a cotton swab, and cells that had migrated or invaded to the lower surface were observed and counted under a microscope.

### RT-qPCR assay

Total RNA was extracted using TRIzol (EP013, ELK Biotechnology), and its concentration was determined using a NanoDrop. The RNA was subsequently reverse-transcribed into cDNA using a reverse transcription kit (EQ003, ELK Biotechnology). qPCR was performed using SYBR Green mix (EQ001, ELK Biotechnology). The reaction system was prepared according to the manufacturer’s instructions and run on a QuantStudio 6 Flex qPCR instrument (Life technologies). Telative gene expression was calculated using the 2^−DDCT^ method. Primer sequences were as follows: β-actin: Forward: 5′- GTC​CAC​CGC​AAA​TGC​TTC​TA-3′, Reverse: 5′- TGC​TGT​CAC​CTT​CAC​CGT​TC-3′; ARAF: Forward: 5′- CGT​CAA​AGT​ATA​CCT​GCC​CAA​C-3′, Reverse: 5′- TGA​TGA​GTC​GGT​AGA​CCA​CAC​AG-3′.

### Western blot assay

Proteins were extracted using RIPA lysis buffer (AS1004, ASPEN) and quantified using a BCA quantification kit (AS1086, ASPEN). After quantification, 5 × loading buffer (AS1011, ASPEN) was added, and samples were denatured at 95 °C for 10 min. Proteins were then separated by SDS-PAGE using a precast gel (LK302, Epizyme) and transferred to a PVDF membrane (IPVH00010, Merck Millipore) using a constant current. Membranes were washed, blocked, and incubated overnight at 4 °C (antibody details are provided in Table 1). After washing, membranes were incubated with HRP-conjugated secondary antibody (AS1107, ASPEN, 1:5000) at room temperature for 2–4 h. Protein bands were visualized using ECL chemiluminescent solution (AS1059, ASPEN) and imaged with a fluorescence imaging system (SCG-W2000, Servicebio).

**Table 1:** Information of primary antibody.

Name	Supplier	Catalog	Dilution
E-cadherin	CST	3,195	1:500
N-cadherin	CST	13,116	1:1000
Cleaved-caspase3	Affinity	AF7022	1:1000
Caspase3	Abcam	ab32351	1:1000
p-p38	CST	4,511	1:500
p38	CST	8,690	1:3000
p-ERK	CST	4,370	1:1000
ERK	CST	4,695	1:3000
p-MEK	CST	9,154	1:1000
MEK	CST	9,126	1:5000
ARAF	Abcam	ab314539	1:1000
β-actin	TDY	TDY051	1:10000

### Statistical analysis

All experiments were performed with at least three independent biological replicates. Data are presented as the mean ± standard deviation (SD). Comparisons between two groups were performed using Student’s t-test, and comparisons among multiple groups were conducted using one-way ANOVA followed by Tukey’s multiple-comparison test. Statistical significance was defined as p<0.05.

## Results

### Analysis of bladder cancer-related genes and pathways

**Figure 1:**
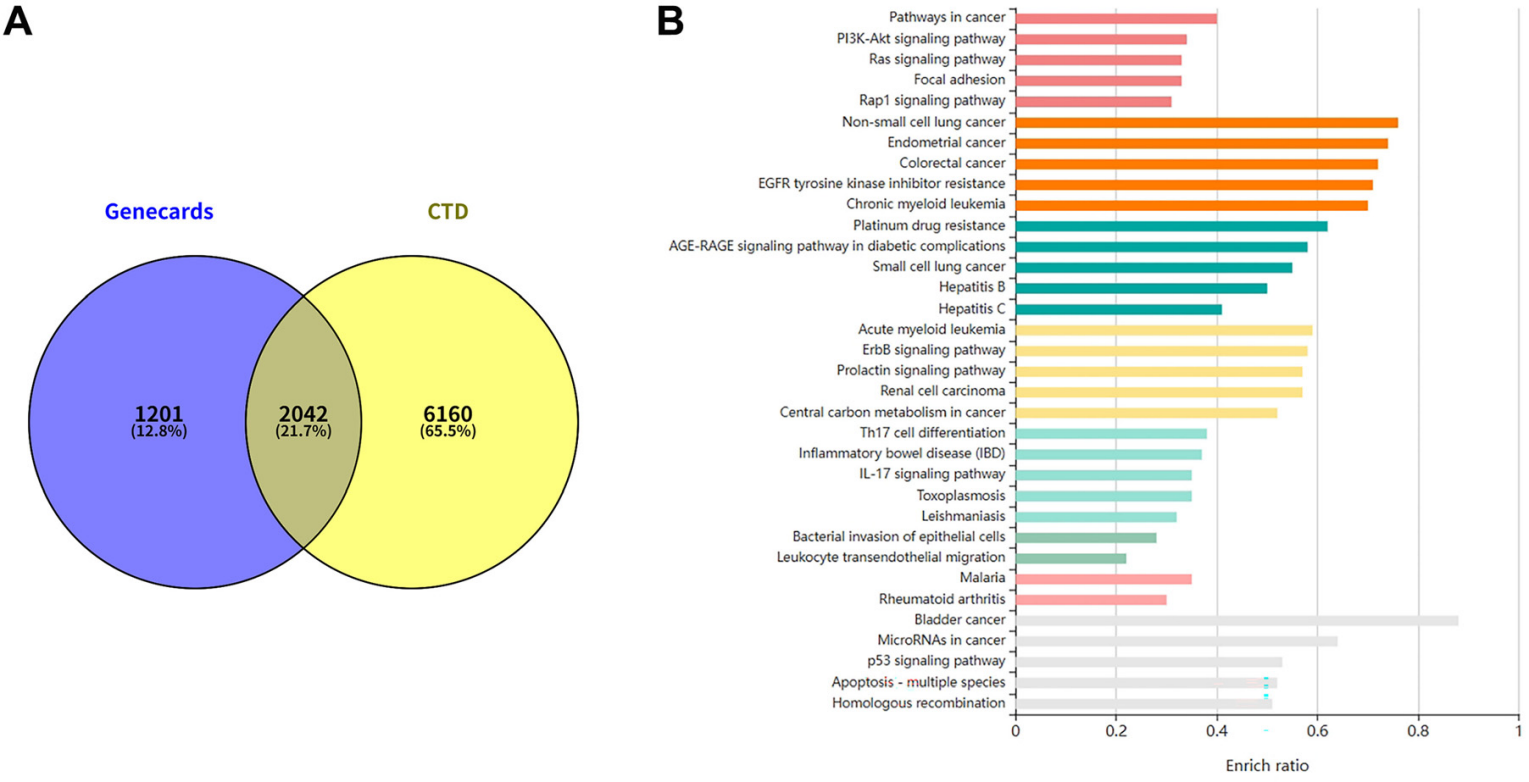
Bioinformatics analysis of bladder cancer-related genes and pathways. (A) Venn diagram showing the intersection of bladder cancer-related genes from the CTD and GeneCards databases. (B) KEGG pathway enrichment analysis of the intersecting genes.

To identify genes and pathways closely associated with bladder cancer, we analyzed the CTD (Supplementary Data 1) and GeneCards (Supplementary Data 2) databases and determined their intersection to obtain bladder cancer-related genes (Figure 1A). A total of 2,042 genes (21.7 %) were shared between the GeneCards and CTD databases, representing the proportion of overlapping genes relative to the total number of genes in both databases (Supplementary Data 3). The intersecting genes were then subjected to KEGG pathway enrichment analysis using the KOBAS database to identify the top pathways (Figure 1B, Supplementary File 1). Among the pathways identified, ARAF was ranked highly in relevance to bladder cancer (Supplementary File 1). Currently, research on ARAF in bladder cancer is limited to omics studies, and its specific role and mechanism have not been reported. Further analysis revealed that, in addition to being enriched in bladder cancer-related pathways, ARAF was also enriched in the MAPK pathway (Supplementary File 1).

### Elevated expression of ARAF in bladder cancer

**Figure 2:**
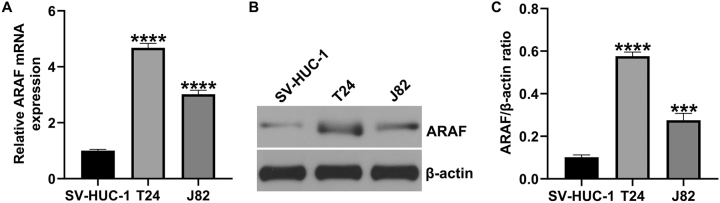
Expression levels of ARAF in bladder cancer cells. (A) mRNA expression of ARAF in different cell lines. (B and C) Protein expression of ARAF in different cell lines. Data are presented as mean ± SD from three independent biological replicates. Statistical analysis was performed using one-way ANOVA. ***p<0.001, ****p<0.0001 vs. SV-HUC-1.

To assess ARAF expression in bladder cancer, we cultured bladder cancer cell lines *in vitro* and measured their expression levels. ARAF expression was significantly higher in T24 and J82 cells compared with SV-HUC-1 cells, with the highest expression observed in T24 cells (Figure 2A–C). These results indicated that ARAF expression was elevated in bladder cancer.

### Inhibition of ARAF expression suppresses malignant proliferation of T24 cells

**Figure 3:**
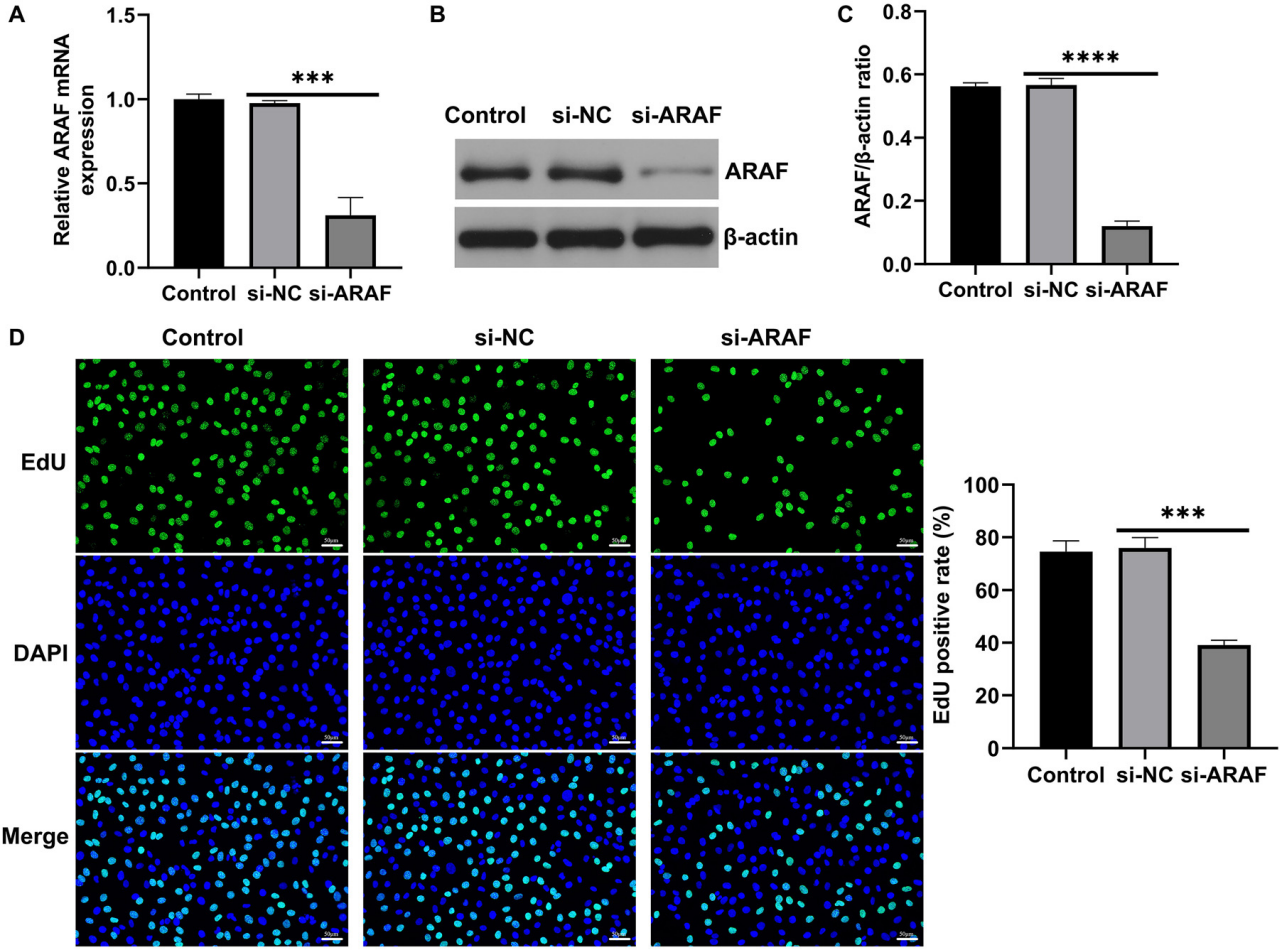
Effect of ARAF on the proliferation of T24 cells. (A) RT-qPCR analysis of si-ARAF transfection efficiency. (B and C) Western blot analysis of si-ARAF transfection efficiency. (D) EdU fluorescence assay showing proliferating cells. Data are presented as mean ± SD from three independent biological replicates. Statistical analysis was performed using one-way ANOVA. ***p<0.001, ****p<0.0001.

**Figure 4:**
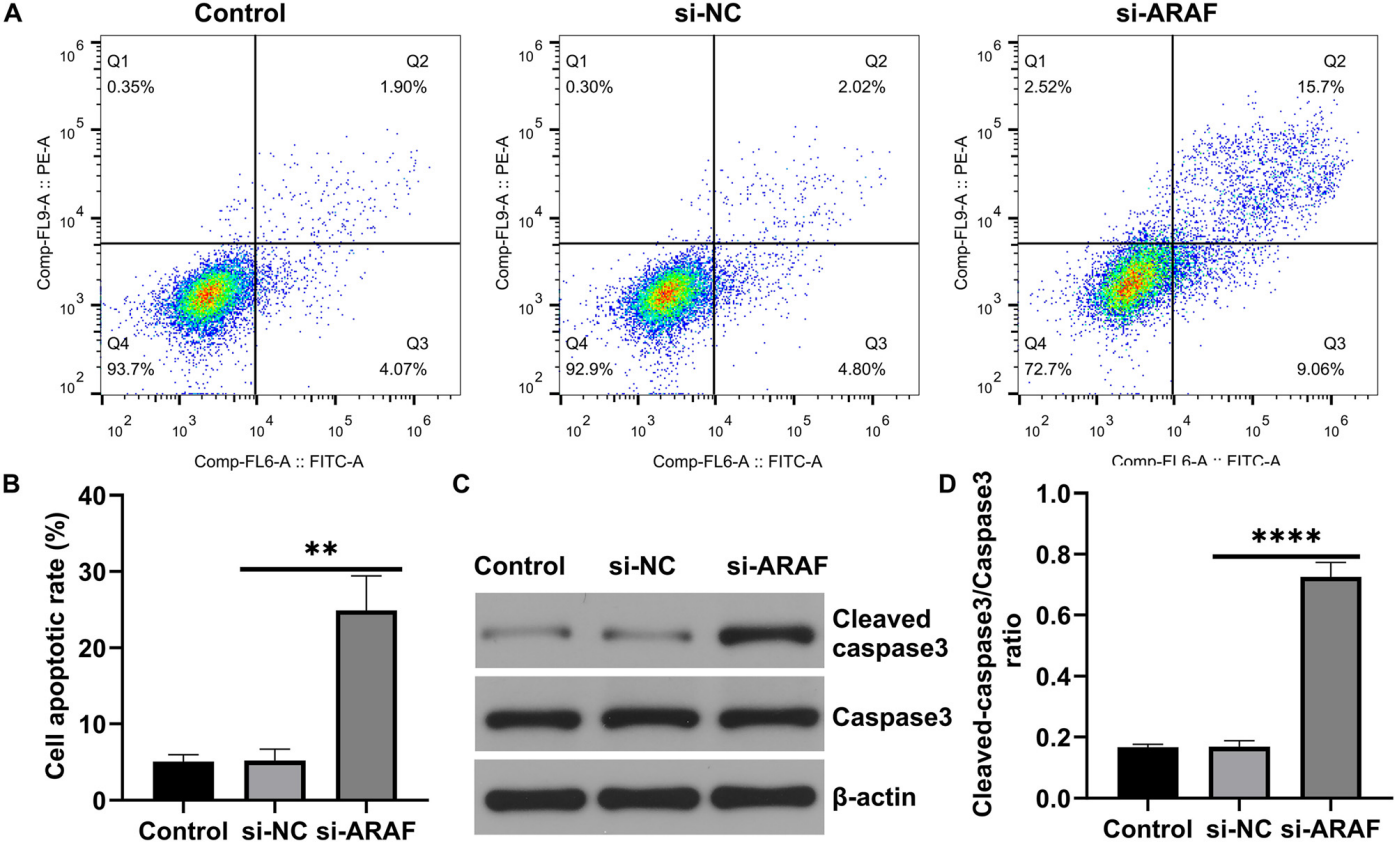
Effect of ARAF on apoptosis of T24 cells. (A and B) Flow cytometry analysis of apoptosis levels. (C and D) Protein expression of cleaved-caspase 3 and total caspase 3. Data are presented as mean ± SD from three independent biological replicates. Statistical analysis was performed using one-way ANOVA. **p<0.01, ****p<0.0001.

To determine the effect of ARAF on the malignant proliferation of T24 cells, si-ARAF and its negative control were constructed and transfected into T24 cells. First, the interference efficiency of si-ARAF was confirmed: transfection with si-ARAF significantly reduced ARAF expression compared with the control and negative control groups (Figure 3A–C). EdU fluorescence results indicated that ARAF inhibition reduced the proportion of EdU-positive cells, suggesting that cell proliferation was inhibited (Figure 3D). Annexin V-FITC/PI double staining showed that silencing ARAF promoted apoptosis in T24 cells (Figure 4A and B). In addition, western blot analysis showed that cleaved-caspase 3 levels were significantly higher in the si-ARAF group compared with the control group (Figure 4C and D). These results indicated that inhibition of ARAF expression could suppress malignant proliferation and promote apoptosis in bladder cancer cells.

### Inhibition of ARAF expression suppresses malignant metastasis of T24 cells

**Figure 5:**
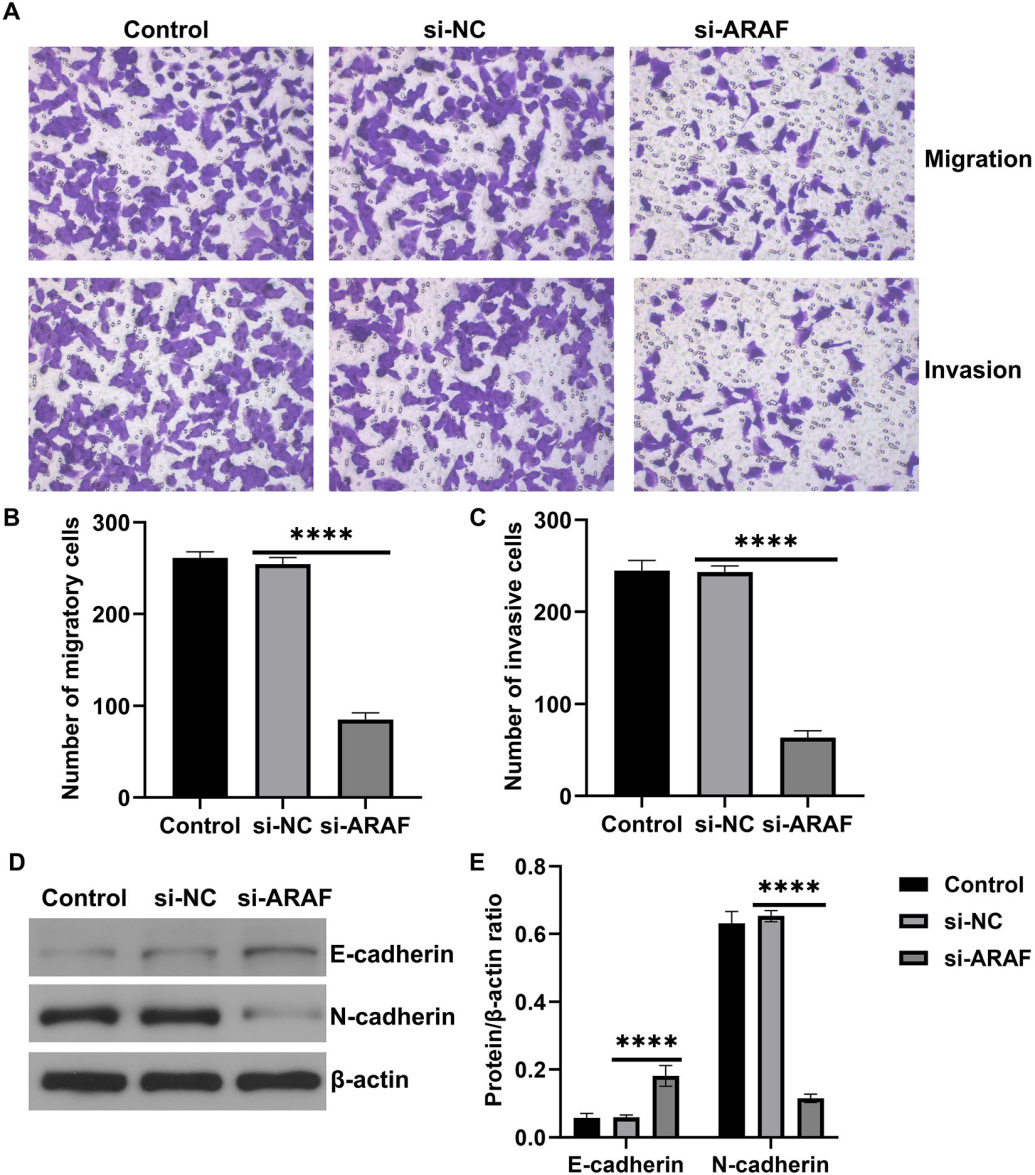
Effect of ARAF on the metastatic capacity of T24 cells. (A and B) Quantification of migrating cells. (A and C) Quantification of invading cells. (D and E) Expression of EMT-related proteins in T24 cells. Data are presented as mean ± SD from three independent biological replicates. Statistical analysis was performed using one-way ANOVA. ****p<0.0001.

Transwell assays were performed to determine the metastatic potential of T24 cells. The results showed that ARAF inhibition significantly reduced cell migration and invasion (Figure 5A–C). Western blot analysis of EMT-related proteins demonstrated that ARAF inhibition promoted E-cadherin expression while decreasing N-cadherin expression (Figure 5D and E). These results indicated that ARAF inhibition could alleviate the malignant metastatic behavior of bladder cancer cells.

### Inhibition of ARAF expression suppresses p38 MAPK pathway activation in T24 cells

**Figure 6:**
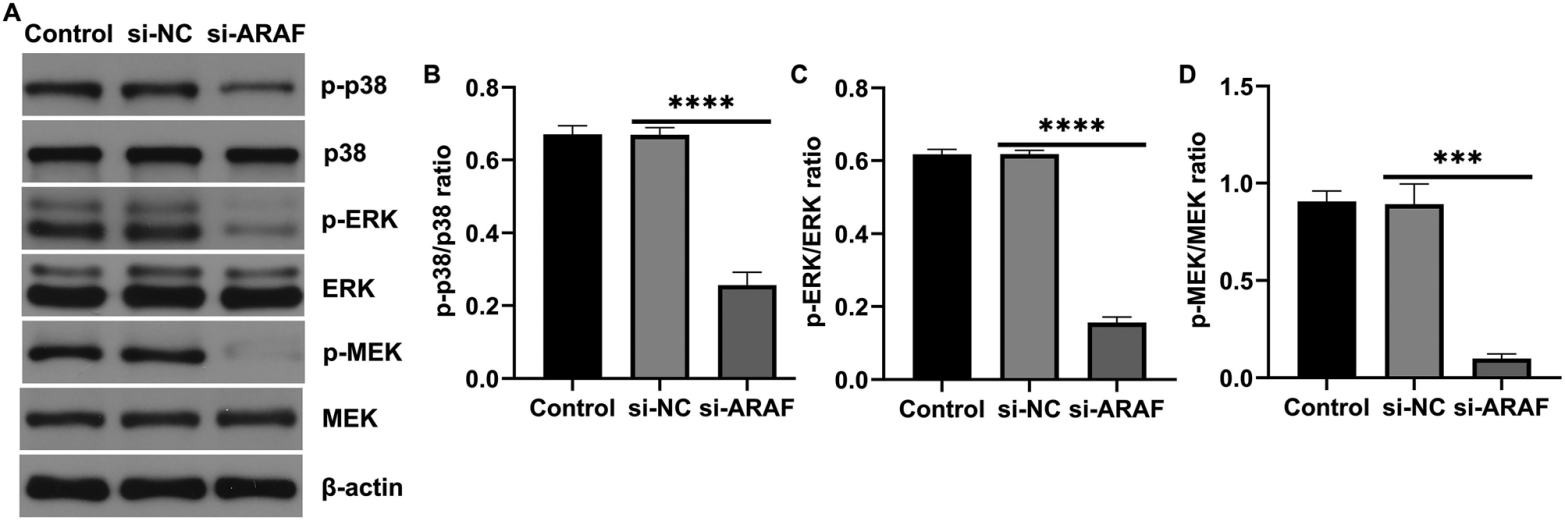
Effect of ARAF on the p38 MAPK pathway in T24 cells. (A) The level of p-p38, p38, p-ERK, ERK, p-MEK, MEK in T24 cells was determined by western blot assay. (B) p-p38/p38 ratio. (C) p-ERK/ERK ratio. (D) p-MEK/MEK ratio. Data are presented as mean ± SD from three independent biological replicates. Statistical analysis was performed using one-way ANOVA. ***p<0.001, ****p<0.0001.

KEGG pathway analysis showed that ARAF is mainly involved in regulating the MAPK pathway. To investigate this, western blot assays were performed to assess the activation of the p38 MAPK pathway. The results showed that ARAF inhibition reduced the phosphorylation levels of p38, ERK1/2, and MEK in T24 cells (Figure 6A–D), suggesting that ARAF promotes p38 MAPK pathway activation.

### ARAF regulates T24 cell proliferation and metastasis through the p38 MAPK pathway

**Figure 7:**
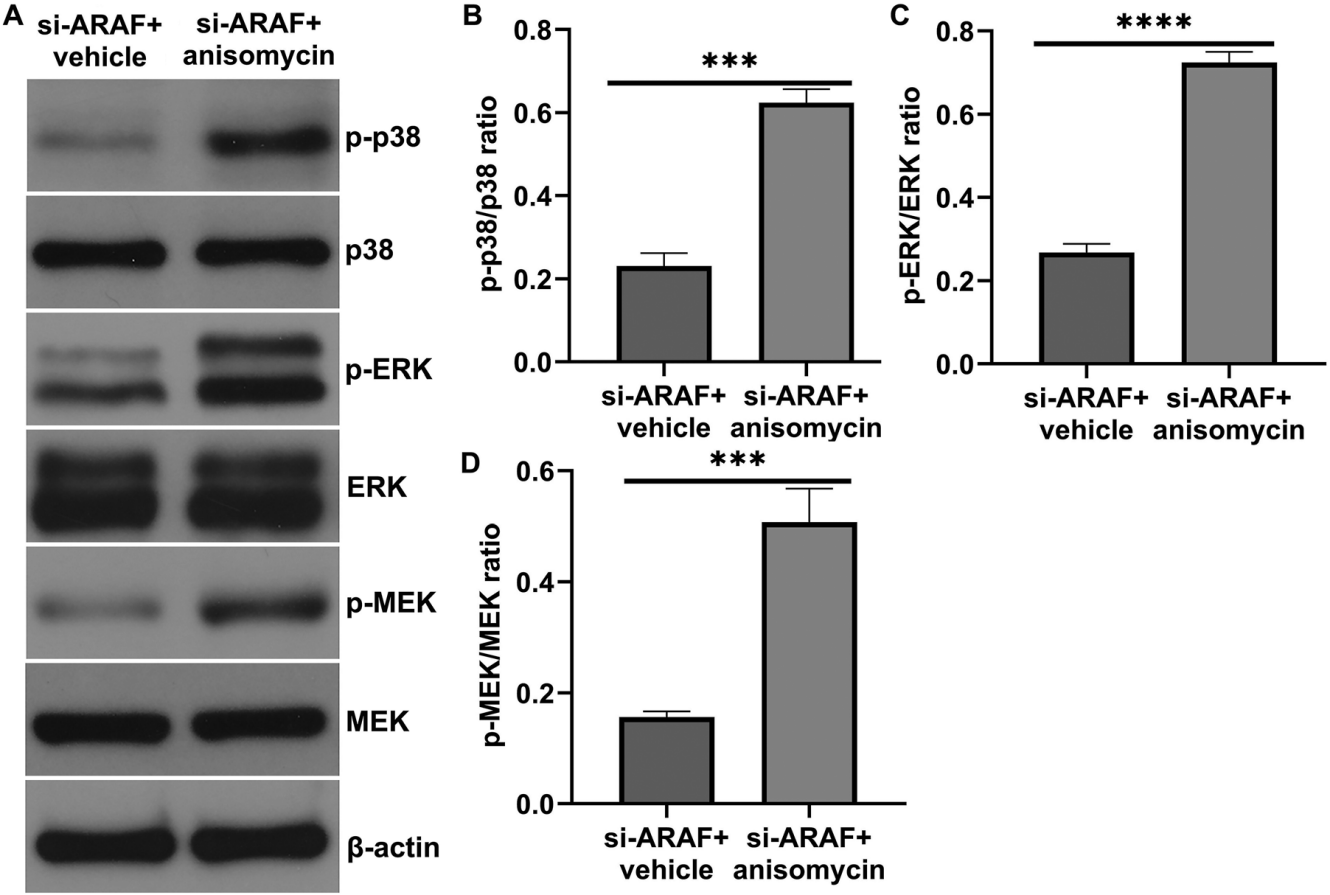
Anisomycin activated p38 MAPK pathway in si-ARAF transfected T24 cells. (A) The level of p-p38, p38, p-ERK, ERK, p-MEK, MEK in T24 cells was determined by western blot assay. (B) p-p38/p38 ratio. (C) p-ERK/ERK ratio. (D) p-MEK/MEK ratio. Data are presented as mean ± SD from three independent biological replicates. Statistical analysis was performed using Student’s t-test. ***p<0.001, ****p<0.0001.

**Figure 8:**
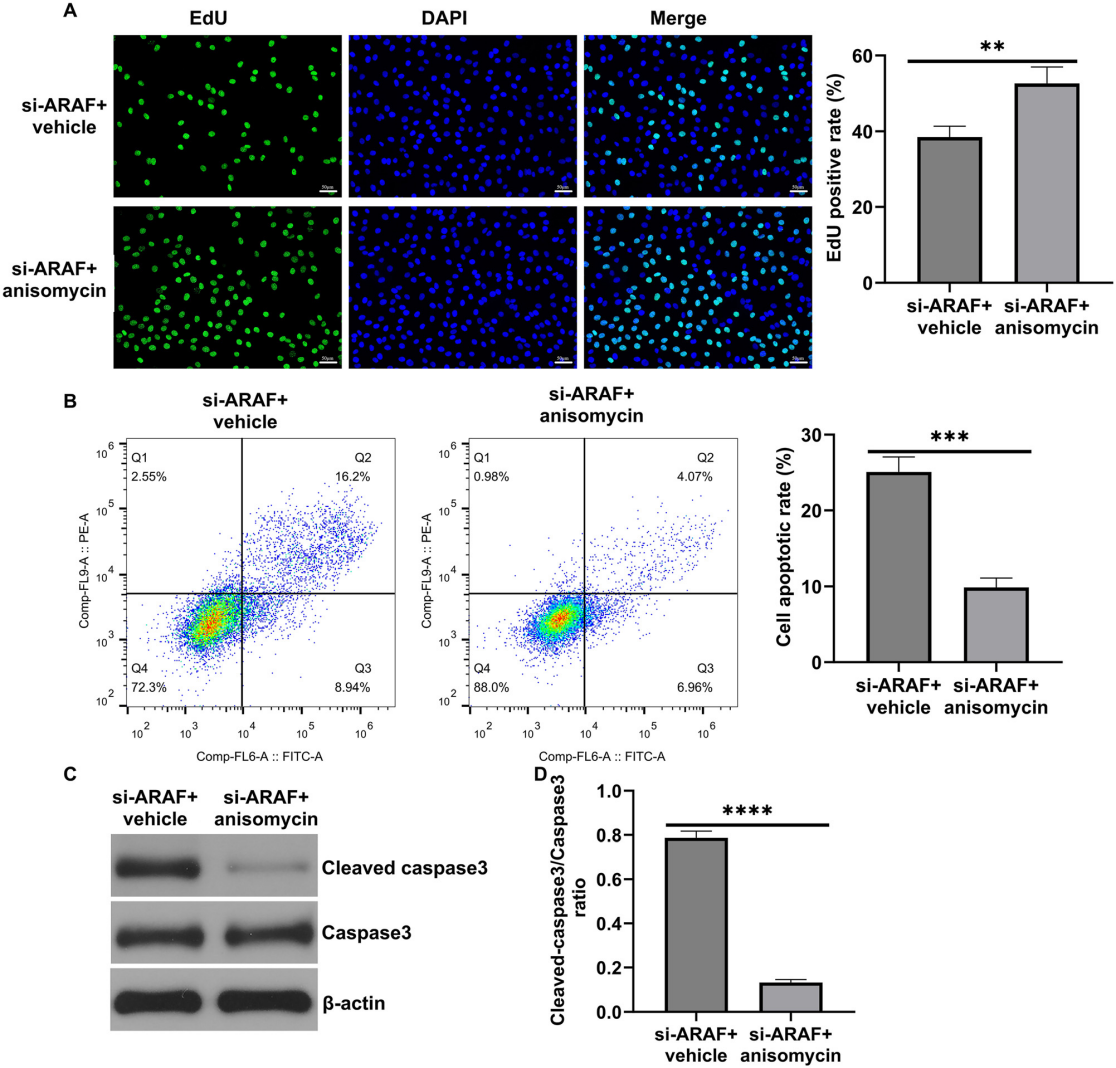
Anisomycin reversed the effect of ARAF on T24 cell proliferation and apoptosis. (A) EdU fluorescence for cell proliferation capacity. (B) The apoptotic rate of T24 cells. (C and D) The protein level of Cleaved-caspase3 and Caspase3. Data are presented as mean ± SD from three independent biological replicates. Statistical analysis was performed using Student’s t-test. **p<0.01, ***p<0.001, ****p<0.0001.

**Figure 9:**
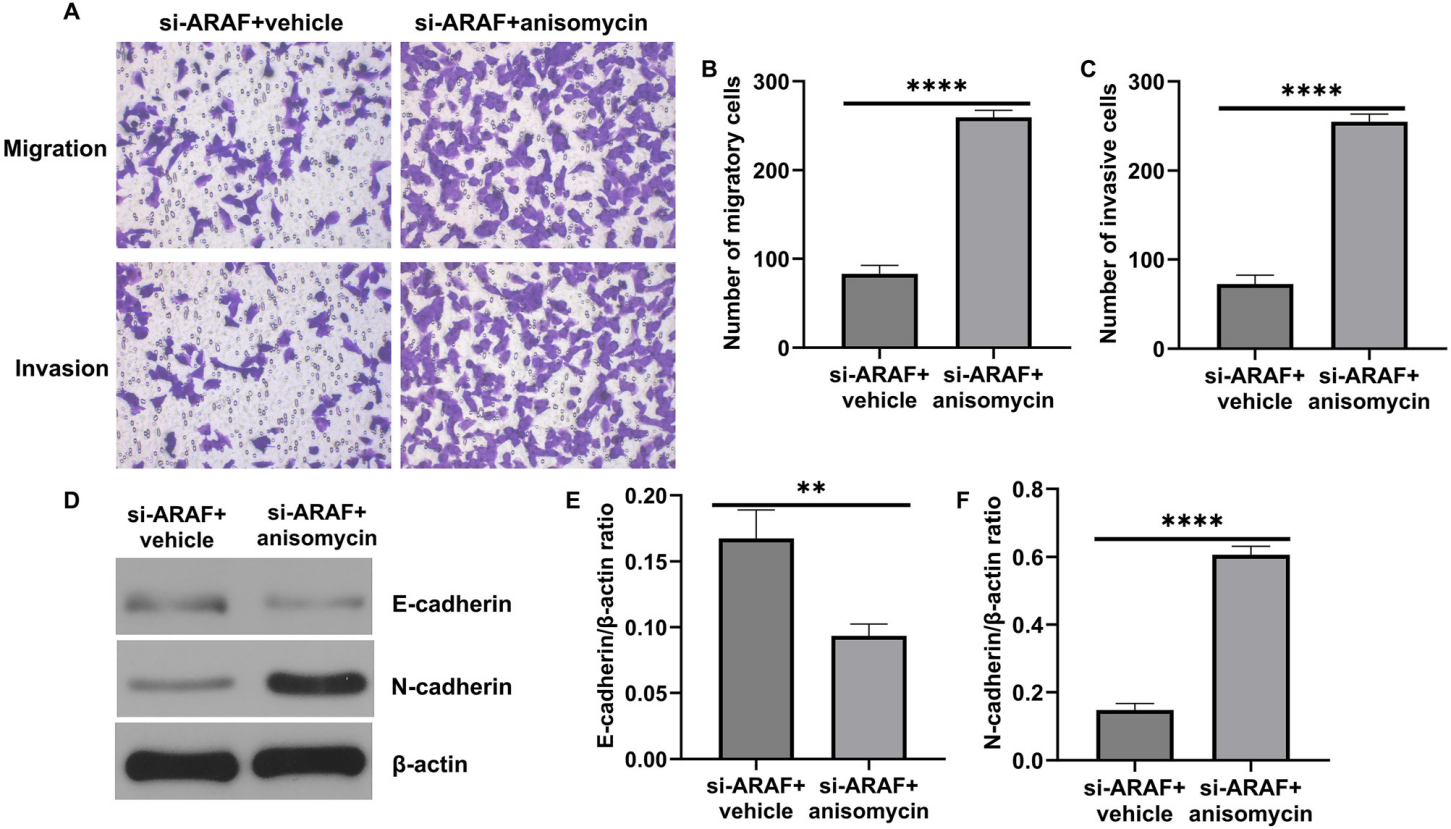
Anisomycin reversed the effect of ARAF on T24 cell metastasis and EMT. (A–C) The migratory and invasive ability of T24 cells. (D–F) The protein level of E-cadherin and N-cadherin. Data are presented as mean ± SD from three independent biological replicates. Statistical analysis was performed using Student’s t-test. **p<0.01, ****p<0.0001.

Among the MAPK family members, p38 MAPK was the focus of in-depth mechanistic studies in this work. Other kinases such as ERK and MEK were only examined for preliminary changes, and their potential roles were not explored further. To investigate whether the p38 MAPK pathway mediates ARAF’s regulation of bladder cancer proliferation and metastasis, anisomycin was used as a pharmacological activator to confirm the involvement of p38 MAPK activation. However, it should be noted that Anisomycin is a broad-spectrum activator of p38 MAPK and may have potential off-target effects. Western blot results showed that anisomycin promoted the phosphorylation of the MAPK pathway in cells (Figure 7A–D). EdU results showed that the proportion of EdU-positive cells was higher in the si-ARAF+anisomycin group compared with the si-ARAF group, indicating that anisomycin partially rescued the proliferation of T24 cells inhibited by ARAF silencing (Figure 8A). Flow cytometry analysis showed that anisomycin treatment markedly reduced apoptosis in cancer cells (Figure 8B). Consistently, western blot analysis of apoptosis-related proteins revealed that anisomycin decreased cleaved-caspase 3 expression (Figure 8C and D). In addition, migration and invasion assays showed that anisomycin promoted cancer cell migration and invasion (Figure 9A–C) and decreased E-cadherin expression while promoting N-cadherin expression (Figure 9D–F). Taken together, these results suggest that ARAF regulates the malignant proliferation and metastasis of bladder cancer cells, at least in part, by regulating the activation of the p38 MAPK pathway.

## Discussion

In the present study, we found that inhibition of ARAF curbs malignant proliferation and metastasis of cancer cells by regulating the p38 MAPK pathway, providing a novel and plausible target for the treatment of bladder cancer.

As medical treatments continue to evolve, several treatment options are available for bladder cancer. The primary treatments include surgery, intravesical instillation therapy, radiotherapy, chemotherapy, immunotherapy, and targeted therapy. However, each method has specific indications and limitations [24], [25], [26]. For example, surgical treatment may affect a patient’s quality of life and psychological state [27], While chemotherapy may cause toxic side effects such as vomiting and hair loss and may result in unpredictable drug resistance [28]. Therefore, precision medicine strategies are an important direction for future research.

As the concept of precision medicine has entered the public eye, the development of biomarkers for the diagnosis and treatment of bladder cancer has gradually become a research hotspot. For example, one study reported that alpha-1,3-mannosyltransferase (**ALG3**) expression was significantly higher in bladder cancer tissues compared with normal tissues, and that its expression was positively correlated with clinical prognosis. Inhibition of ALG3 suppressed the proliferation and migration of bladder cancer cell lines, indicating that ALG3 plays a key role in bladder cancer development [29]. Recently, family with sequence similarity 171 member B (FAM171B) was shown to promote tumor-associated macrophage infiltration and polarization by stabilizing cytoplasmic vimentin and enhancing CCL2 splicing, thereby significantly promoting the growth and metastasis of bladder cancer. This finding provides a theoretical basis for the use of FAM171B as a diagnostic and therapeutic biomarker for bladder cancer [30]. In our study, we found that ARAF expression was elevated in bladder cancer, and that inhibition of ARAF expression reduced malignant proliferation and metastasis of bladder cancer cells. In addition, ARAF knockdown significantly inhibited EMT cancer cells, as evidenced by enhanced E-cadherin expression and reduced N-cadherin expression. Notably, our preliminary observations also revealed that ARAF knockdown significantly suppressed the phosphorylation of MEK and ERK, in addition to p38 MAPK; while the present study focused on the in-depth mechanistic exploration of the ARAF-p38 MAPK axis, this finding further highlights ARAF as a central upstream regulator of MAPK signaling in bladder cancer. These results collectively suggested that ARAF may serve as a potential target for the treatment and diagnosis of bladder cancer.

Previous studies have reported ARAF as a possible biomarker in various cancers [10], [11], [12], and our findings identified ARAF as a novel regulator in bladder cancer, which functions distinctly from previously reported biomarkers such as ALG3 and FAM171B. ALG3 is mainly involved in glycosylation modification and protein processing, while FAM171B contributes to cell adhesion and cytoskeleton organization. In contrast, ARAF acts as a critical kinase that directly governs the p38 MAPK signaling cascade, representing an upstream regulatory node rather than a structural or metabolic effector. Notably, we also observed that ARAF knockdown significantly inhibited MEK and ERK phosphorylation (not further studied in this work), which further supports ARAF as a key upstream regulator of MAPK signaling. This mechanistic difference makes ARAF a functionally complementary target to ALG3 and FAM171B, providing a new intervention direction for bladder cancer.

Aberrant activation of the p38 MAPK pathway had been strongly associated with cancer development in previous studies [31]. In bladder cancer, Thi et al. reported that CD46 overexpression enhanced MMP9 expression in multiple bladder cancer cell lines and, through activation of the p38 MAPK pathway, increased cell migration and invasion [32]. Zhi et al. demonstrated that deletion of FGF6 inhibited p38 MAPK pathway activation and reduced lactate production, glucose uptake, and expression of angiogenic factors and glycolytic enzymes in bladder cancer cells [33]. In our study, we found that ARAF inhibition blocked p38 MAPK pathway activation. Conversely, treatment with anisomycin, a p38 MAPK pathway activator, restored pathway activity and reversed the suppression of proliferation and metastasis induced by ARAF knockdown. Together with previous studies, these results indicated that the p38 MAPK pathway is an important signaling pathway driving malignant progression in bladder cancer, and ARAF is a key upstream regulator of this pathway. However, it remains unclear whether ARAF interacts directly with p38 MAPK components; this question, which could be addressed by co-IP assays, would further confirm the direct regulatory relationship between ARAF and p38 MAPK.

Compared with existing MAPK inhibitors (mostly targeting MEK/ERK or non-selective p38 inhibitors, which often cause drug resistance or off-target effects), ARAF targeting is more advantageous, as it regulates both p38 and MEK/ERK pathways (our preliminary observation) and may avoid these limitations. In addition, ARAF inhibition has potential in combination therapy: combining it with chemotherapy (e.g., cisplatin) may overcome drug resistance, and combining it with immunotherapy may enhance anti-tumor immunity, which deserves further exploration.

Several limitations of this study should be acknowledged. First, all experiments were performed using bladder cancer cell lines (T24 and J82) under *in vitro* conditions. Clinical samples, such as human bladder cancer tissues for immunohistochemical analysis, were not included in the present study. Therefore, the clinical relevance of ARAF as a potential diagnostic biomarker or therapeutic target for bladder cancer remains to be further validated. Future studies using large-scale clinical cohorts and *in vivo* tumor models are warranted to confirm the expression pattern and clinical significance of ARAF in bladder cancer. Another potential limitation of this study is that Anisomycin, a broad p38 MAPK activator, was used for functional rescue assays. Although this approach supported the role of p38 MAPK in the ARAF-mediated phenotype, it is not entirely specific to the ARAF-p38 axis. In future studies, direct rescue experiments via re-expression of ARAF in ARAF knockdown cells will be performed to more rigorously confirm the causal regulatory relationship between ARAF and p38 MAPK signaling. Additionally, due to time and resource constraints, we were unable to perform co-IP assays to investigate whether ARAF interacts directly with p38 MAPK components; this will be a focus of our future research to clarify the direct molecular interaction underlying the ARAF-p38 MAPK regulatory axis. Furthermore, the mechanism by which ARAF regulates MEK/ERK pathway needs further investigation.

Taken together, the present study demonstrated that ARAF regulates bladder cancer cell proliferation and metastasis by modulating the p38 MAPK pathway. This study provides a novel potential target for the diagnosis and treatment of bladder cancer.

## Conclusions

Our results demonstrated that ARAF expression is closely associated with the malignant proliferation and metastasis of bladder cancer cells, and that this regulation is mediated through activation of the p38 MAPK pathway. However, this study has some limitations. Future studies will involve clinical samples and xenograft tumor models to further clarify the role of ARAF in bladder cancer, providing a stronger foundation for its potential use as a biomarker and for the development of precise targeted therapies.

## Supplementary Material

Supplementary Material

Supplementary Material

Supplementary Material

Supplementary Material

Supplementary Material
