# Supplementary figures and images for: ARAF regulates malignant progression of bladder cancer through the p38MAPK pathway
Source: Open Med (Wars). 2026 May 26;21(1):20261422. doi: 10.1515/med-2026-1422 (PMC13210402; doi:10.1515/med-2026-1422)

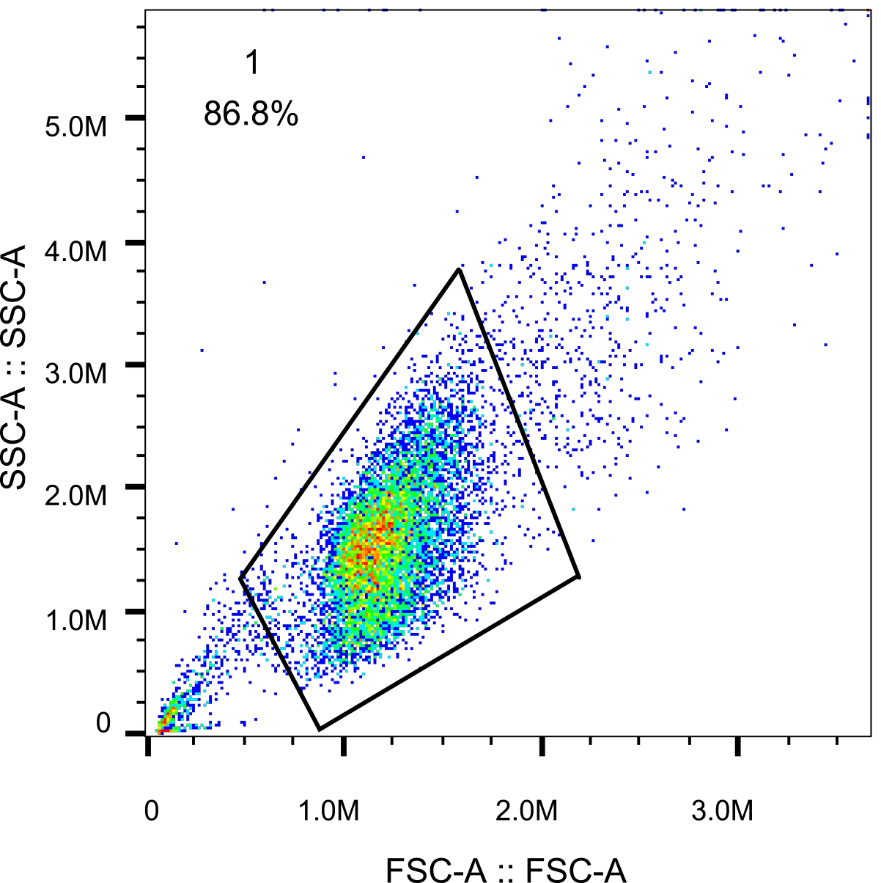


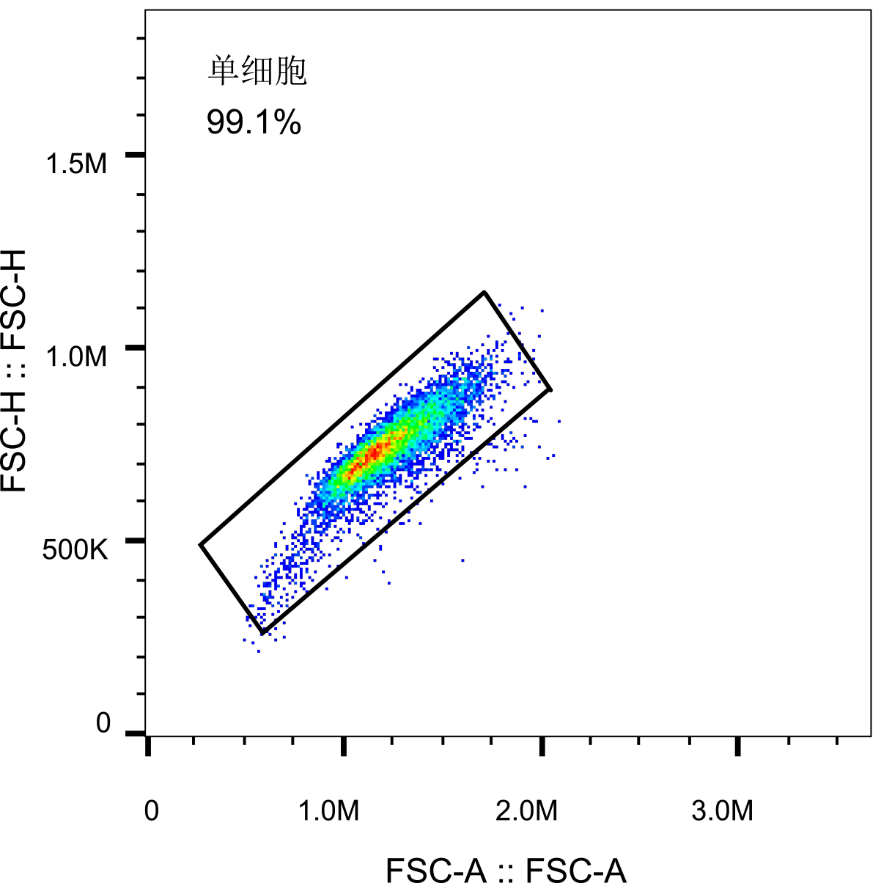


**The gating strategy for the flow cytometry analysis**

Supplement: Supplementary file 3 — Supplementary Material [file j_med-2026-1422_suppl_003.docx]
